# Likelihood of Treatment in a Coronary Care Unit for a First-Time Myocardial Infarction in Relation to Sex, Country of Birth and Socioeconomic Position in Sweden

**DOI:** 10.1371/journal.pone.0062316

**Published:** 2013-04-25

**Authors:** Dong Yang, Stefan James, Ulf de Faire, Lars Alfredsson, Tomas Jernberg, Tahereh Moradi

**Affiliations:** 1 Institute of Environmental Medicine, Division of Cardiovascular Epidemiology, Karolinska Institutet, Stockholm, Sweden; 2 Department of Medical Sciences, Cardiology, Uppsala University, Uppsala, Sweden; 3 Department of Medicine, Section of Cardiology, Huddinge, Karolinska Institutet, Stockholm, Sweden; 4 Centre for Epidemiology and Social Medicine, Health Care Services, Stockholm County Council, Stockholm, Sweden; Tehran University of Medical Sciences, Islamic Republic of Iran

## Abstract

**Objective:**

To examine the relationship between sex, country of birth, level of education as an indicator of socioeconomic position, and the likelihood of treatment in a coronary care unit (CCU) for a first-time myocardial infarction.

**Design:**

Nationwide register based study.

**Setting:**

Sweden.

**Patients:**

199 906 patients (114 387 men and 85,519 women) of all ages who were admitted to hospital for first-time myocardial infarction between 2001 and 2009.

**Main outcome measures:**

Admission to a coronary care unit due to myocardial infarction.

**Results:**

Despite the observed increasing access to coronary care units over time, the proportion of women treated in a coronary care unit was 13% less than for men. As compared with men, the multivariable adjusted odds ratio among women was 0.80 (95% confidence interval 0.77 to 0.82). This lower proportion of women treated in a CCU varied by age and year of diagnosis and country of birth. Overall, there was no evidence of a difference in likelihood of treatment in a coronary care unit between Sweden-born and foreign-born patients. As compared with patients with high education, the adjusted odds ratio among patients with a low level of education was 0.93 (95% confidence interval 0.89 to 0.96).

**Conclusions:**

Foreign-born and Sweden-born first-time myocardial infarction patients had equal opportunity of being treated in a coronary care unit in Sweden; this is in contrast to the situation in many other countries with large immigrant populations. However, the apparent lower rate of coronary care unit admission after first-time myocardial infarction among women and patients with low socioeconomic position warrants further investigation.

## Introduction

Treatment in coronary care units (CCU) for myocardial infarction (MI) is suggested to be associated with improved prognosis [1]. A limited number of studies have reported inequalities in sex, ethnicity and socioeconomic position (SEP) regarding admission to CCU and treatment after MI [2], [3], [4], [5], [6], [7]. Although, reduction in such inequalities over timehas been reported [8], to our knowledge, there is no information available for immigrants by specific country of origin and for men and women separately.

Although care of MI patients in Sweden has significantly improved over the last fifteen years [9], [10], we recently observed that men and low educated patients, independent of country of birth, had increased risk of fatality after day 28 of first time MI diagnosis [11]. If sex, social inequalities and country of birth are proved to affect access to treatment in a CCU and the care of MI patients, they have important implications in the management of care of these patients.

In this observational retrospective study, using nationwide registration of MI and treatment in CCU in Sweden, we identified all patients with first-time MI hospitalized between 2001 and 2009, and examined whether treatment at a CCU was associated with patients’ ethnic, social, demographic and clinical background as well as with hospital characteristics.

## Methods

### Databases

We used data from the Migration and Health Cohort (M&H Co) built by linkage between several Swedish national registers. The data used in this study was part of M&H Co. which included 1) The National Patient Register including the in-patient register established in 1964 which contains information on hospitalized patients and covers the whole of Sweden since 1987 from which we retrieved information about MI and other medical conditions. 2) The Register of Information and Knowledge about Swedish Heart Intensive Care Admissions (RIKS-HIA) which records data on MI patients admitted to hospital CCUs. 3) The Total Population Register where we retrieved demographic variables including country of birth and emigration or immigration. 4) The Swedish Population and Housing Census and Longitudinal Integration Database for Health Insurance and Labor Market studies which includes data regarding education level. 5) The Cause of Death Register which provides information on date of death and causes of death.

Linkages have been completed by Statistics Sweden and the National Board of Health and Welfare using the unique Swedish personal identity number.

### Study Population

All men and women living in Sweden and diagnosed with non-fatal first-time MI (ICD 10th revision (ICD-10) code: I21) registered in the National Patient Register between 2001 and 2009 were included. The study population thus comprised 114,387 men (13,903 foreign born) and 85,519 women (9,601 foreign born).

### Classification of Outcome Variables

Of a total of 199,906 patients who were admitted to hospital after their first-time MI, we identified 120,609 patients received treatment at a CCU and 79,297 who did not. If a patient was admitted to the CCU more than once during the same period of hospitalization, this was treated as one CCU event.

### Classification of Main Exposure Variables

#### Country of birth

Individuals born in Sweden were classified as Sweden-born; those born outside Sweden were classified as foreign-born. For foreign-born individuals, the countries of birth were classified into six broad geographical regions: Africa, Asia, Europe, Latin-America, Northern America, and Oceania, and then sub-grouped according to the United Nations classification and further to individual birth countries. We reported individual data only from countries with five or more MI cases. Countries with less than five hospital admissions due to MI were grouped together, by continent, as “other”. (Table S1 & S2 in File S1).

#### SEP

We used “highest level of education achieved” as a proxy for SEP. We divided level of education into four categories: low (0–9 years), medium (10–12) years, high (more than 12 years), and unknown.

### Other Co-variables

Age at diagnosis of first-time MI was categorized into 13 strata each of 5 years (less than 35, 35–39, 40–44… 85–89, and above 90) due to nonlinear relationship with the outcome. We divided the total study period into three arbitrary time periods: 2001–2003, 2004–2006, and 2007–2009. Additional co-variables considered in the analysis were medical conditions including diabetes (ICD 9th revision (ICD-9): 250; ICD-10: E10–E14), hypertension (ICD-9: 401; ICD-10: I10), and hyperlipidemia (ICD-9: 272; ICD-10: E78), as well as a history of stroke (ICD-9: 430–438; ICD-10: I60–I90), heart failure (ICD-9: 428; ICD-10: I50), angina (ICD-9: 413; ICD-10: I20), atrial fibrillation (ICD-9: 427D; ICD-10: I48), pulmonary embolism (ICD-9: 415B; ICD-10: I26), chronic obstructive lung disease (ICD-9: 490–496; ICD-10: J44), and cancer (ICD-9: 140–239; ICD-10: C00–D48). All medical conditions were verified for a fixed period of 14 years. Moreover, to exclude hospital variations in the availability of CCUs that might have an impact on the possibility of admission, we further classified hospitals into two categories: with and without CCU facilities (yes/no).

### Statistical Analysis

Using multivariable logistic regression, we calculated odds ratios (ORs) and their 95% confidence intervals (CIs) estimated by maximum likelihood tests to explore whether treatment at a CCU among patients with first-time MI was associated with subjects’ country of birth, sex, SEP, age and medical conditions. All analyses were performed for men and women separately. In the first model, we only included age. The effect of other potential confounding factors such as year of diagnosis, medical conditions, and availability of CCU facilities in the hospital was tested in multivariable models. We used SAS software version 9.2. A P-value of <0.05 was considered statistically significant and all tests were two sided.

We repeated the analysis confined to: 1) only first-time MI patients who survived the first day of hospitalization, to eliminate biases caused by cases of extremely severe hospitalized MI; and 2) only foreign-born individuals who had lived in Sweden for more than 14 years (10,403 males and 6,758 females) as well as Sweden-born individuals, to reduce the impact of variation in duration of residence in Sweden and to remove any plausible misclassification of first time MI in foreign-born patients. 3) Only including patients treated initially at hospitals with a CCU (90,186 males and 66,715 females). The results for both these secondary analyses were similar to the findings of the main analyses without restrictions (data not shown).

### Ethics Statement

The study was approved by regional board of the ethical committee in Stockholm (Regionala etikprövningsnämnden i Stockholm; Dnr: 2009/587-32). According to Personal Information Act came into force in 1998 in Sweden (Personuppgiftslagen), written informed consent from the participants is not required since the study is based on national registers and none participant can be identified in the study.

## Results

The characteristics of patients with first-time MI who were admitted to hospital and the proportion treated at a CCU, by sex and country of birth (within or outside Sweden) are shown in Table 1. The total number of first-time MI patients who were admitted to hospital decreased from 23,356 in 2001 to 19,385 in 2009. This decreasing trend was only observed for Sweden-born patients, whereas a slight increase among foreign-born patients was found. Nevertheless, the overall proportion of patients treated in CCU increased over time for all groups. The proportion of women treated at a CCU was 13% less than in men; this lower proportion was consistently observed among both foreign-born and Sweden-born MI patients.

**Table 1:** Characteristics of patients with first-time myocardial infarction (MI) living in Sweden between 2001 and 2009, by sex, country of birth, and study year.

	Men						Women					
	Sweden born			Foreign born			Sweden born			Foreign born		
	2001–2003	2004–2006	2007–2009	2001–2003	2004–2006	2007–2009	2001–2003	2004–2006	2007–2009	2001–2003	2004–2006	2007–2009
**Total number of cases**	35,552	33,833	31,099	4,371	4,525	5,007	27,223	25,472	23,223	3,131	3,189	3,281
**Access to the CCU** **(%)**	61.04	64.37	69.89	67.35	71.16	76.81	49.87	50.88	56.88	54.52	57.13	64.31
**Mean age (years)**	71.57	71.56	71.10	64.60	64.77	64.54	77.86	78.22	77.88	72.95	73.36	73.44
±**SD**	±12.43	±12.52	±12.64	±12.74	±12.73	±12.71	±11.14	±11.48	±11.95	±11.64	±11.61	±12.08
**Education level (%)**												
Less than 9 years	52.25	49.69	46.01	38.14	35.67	34.15	61.19	61.07	56.69	47.94	48.17	43.52
9 to 12 years	23.87	24.84	25.50	24.62	24.35	25.42	22.00	24.25	27.77	21.85	24.02	24.17
More than 12 years	20.52	23.91	27.76	28.37	32.44	32.69	7.98	10.37	13.58	12.39	13.23	17.95
Unknown	3.36	1.56	0.74	8.88	7.54	7.73	8.83	4.31	1.97	17.82	14.58	14.36
**Medical conditions (%)**												
Diabetes	23.65	23.00	22.16	27.02	27.29	26.44	23.66	23.11	21.94	30.37	29.76	28.35
Hyperlipidemia	24.92	23.77	27.86	34.29	30.52	33.09	16.20	16.22	19.60	23.76	24.24	26.79
Hypertension	37.65	45.02	52.71	39.53	44.49	51.17	43.20	51.74	61.17	48.13	57.13	63.64
Stroke	13.61	13.36	12.38	11.12	10.48	9.23	15.32	15.29	14.67	13.19	13.70	14.45
Heart failure	11.43	10.88	10.11	10.41	9.88	8.75	15.98	15.19	14.81	15.87	16.15	15.94
Angina	16.82	16.81	16.84	16.88	18.76	17.06	16.99	16.88	16.64	19.51	20.63	20.94
Atrial fibrillation	10.05	11.39	12.09	7.34	7.65	8.31	13.09	14.22	15.12	10.86	12.89	13.59
Pulmonary embolism	0.96	1.19	1.29	0.59	0.86	0.78	1.32	1.69	1.96	1.34	1.13	1.13
COLγ	5.66	5.47	5.29	5.99	6.48	5.19	6.62	6.78	7.05	8.27	8.69	8.75
Cancer	16.62	21.00	24.37	12.65	15.71	18.29	18.32	22.46	26.79	17.82	19.57	23.90

γ Chronic obstructive lung disease.

Overall, foreign-born first-time MI patients were younger (mean age±SD: men, 64.63±12.73 years; women, 73.25±11.78 years) than their Sweden-born counterparts (men, 71.42±12.53 years; women, 77.99±11.51 years). The mean age at diagnosis remained almost constant throughout the study period (Table 1).

There was no evidence of an overall association between country of birth and the likelihood of treatment at a CCU (Table 2), but a lower access was evident for women than for men (Table 3). Both Sweden-born and foreign-born female patients had around 10–20% lower odds of being treated at a CCU compared with Sweden-born male patients, even after adjusting for level of education, medical conditions, and hospital characteristics (Table 3). Analysis among foreign-born patients showed similar results. The proportion of foreign-born women being treated at a CCU was about 20–30% lower than that for foreign-born men (data not shown).

**Table 2:** Odds ratio (OR) and 95% confidence interval (CI) of access to a coronary care unit (CCU) after first-time myocardial infarction by sex, country of birth, and year of diagnosis in patients living in Sweden between 2001 and 2009.

	Men				Women			
	Access to CCU yes/no	OR* (95% CI)	OR** (95% CI)	OR*** (95% CI)	Access to CCU yes/no	OR* (95% CI)	OR** (95% CI)	OR*** (95% CI)
**Country of birth**								
Foreign born	10,010/3,893	1.00 (0.96–1.05)	1.01 (0.97–1.06)	0.97 (0.93–1.02)	5,639/3,962	1.03 (0.98–1.08)	1.02 (0.97–1.07)	1.01 (0.96–1.06)
Sweden born	65,215/35,269	1	1	1	39,745/36,173	1	1	1
**Education**								
Less than 9 years	34,217/20,468	**0.86 (0.83–0.89)**	**0.89 (0.86–0.92)**	**0.93 (0.89–0.96)**	25,368/24,474	**0.88 (0.84–0.93)**	**0.92 (0.87–0.96)**	0.95 (0.90–1.00)
9 to 12 years	19,045/9,226	**0.85 (0.82–0.88)**	**0.86 (0.83–0.90)**	**0.90 (0.86–0.93)**	12,351/8,505	**0.93 (0.88–0.98)**	0.95 (0.90–1.00)	0.97 (0.92–1.03)
More than12 years	20,682/7,681	1	1	1	5,990/3,377	1	1	1
Unknown	1,281/1,787	**0.67 (0.62–0.73)**	**0.73 (0.67–0.80)**	**0.75 (0.69–0.82)**	1,675/3,779	**0.69 (0.64–0.75)**	**0.76 (0.70–0.83)**	**0.80 (0.73–0.87)**
**Year of diagnosis**								
2001–2003	24,646/15,277	**0.65 (0.63–0.67)**	**0.66 (0.64–0.68)**	**0.65 (0.63–0.68)**	15,282/15,072	**0.70 (0.67–0.72)**	**0.71 (0.68–0.73)**	**0.72 (0.70–0.75)**
2004–2006	24,998/13,360	**0.77 (0.74–0.79)**	**0.77 (0.75–0.80)**	**0.78 (0.75–0.81)**	14,783/13,878	**0.76 (0.73–0.79)**	**0.76 (0.74–0.79)**	**0.78 (0.75–0.80)**
2007–2009	25,581/10,525	1	1	1	15,319/11,185	1	1	1

* Adjusted for age at diagnosis.

** Mutually adjusted for other variables in the Table.

*** dditionally adjusted for medical conditions, and availability of CCU facilities.

**Bold** numbers indicate statistically significant ORs.

**Table 3:** Odds ratio (OR) and 95% confidence interval (CI) of access to a coronary care unit (CCU) by sex, country of birth, and year of study.

	Men				Women					
	Sweden–born	Foreign–born			Sweden–born			Foreign–born		
		OR (95% CI)			OR (95% CI)			OR (95% CI)		
Stepwise models	Reference foreach period	2001–2003	2004–2006	2007–2009	2001–2003	2004–2006	2007–2009	2001–2003	2004–2006	2007–2009
Model 1	1	0.97 (0.90–1.04)	0.99 (0.92–1.06)	1.01 (0.94–1.09)	**0.88 (0.85–0.91)**	**0.81 (0.79–0.84)**	**0.85 (0.81–0.88)**	**0.79 (0.73–0.85)**	**0.77 (0.71–0.83)**	**0.85 (0.79–0.92)**
Model 2	1	0.98 (0.91–1.05)	1.01 (0.94–1.09)	1.02 (0.95–1.10)	**0.89 (0.86–0.92)**	**0.82 (0.79–0.85)**	**0.86 (0.82–0.89)**	**0.82 (0.76–0.88)**	**0.81 (0.75–0.88)**	**0.88 (0.81–0.96)**
Model 3	1	0.99 (0.93–1.07)	1.05 (0.98–1.13)	1.05 (0.97–1.13)	**0.87 (0.84–0.90)**	**0.80 (0.77–0.83)**	**0.83 (0.79–0.86)**	**0.82 (0.76–0.89)**	**0.81 (0.75–0.88)**	**0.89 (0.82–0.97)**
Model 4	1	0.99 (0.93–1.07)	1.05 (0.97–1.13)	1.05 (0.97–1.13)	**0.87 (0.84–0.90)**	**0.80 (0.77–0.83)**	**0.83 (0.79–0.86)**	**0.82 (0.76–0.89)**	**0.81 (0.75–0.88)**	**0.90 (0.83–0.98)**

All odds ratios were compared with Sweden–born men in the corresponding time period.

Model 1 includes: age at diagnosis in 5–year categories.

Model 2 includes: variables in model 1+ education level.

Model 3 includes:variables in model 2+ medical conditions.

Model 4 includes: variables in model 3+ availability of a CCU.

**Bold** numbers indicate statistically significant ORs.

We observed a lower access to the CCU among patients with a low level of education compared with more highly educated first-time MI patients (Table 2); a stratified analysis by country of birth revealed similar results. While education had a borderline significant effect on the likelihood of being treated at a CCU among women, regardless of country of birth, the OR for treatment at a CCU was 6% lower (OR 0.94; 95% CI 0.90–0.97) among men with a low level of education born within Sweden and 14% lower (OR 0.86; 95% CI 0.78–0.95) among those born outside Sweden, compared with highly educated Sweden-born and foreign-born men, respectively.

The multivariable odds of being admitted to a CCU increased by around 30% over the observed 9-year period regardless of sex (Table 2) and country of birth (data not shown).

The adjusted OR values for patients from individual countries were consistent with the results obtained when foreign-born patients as a whole were compared with those born in Sweden. The majority of foreign-born patients showed no statistically significant differences in access to treatment in a CCU compared with Sweden-born patients. Exceptions to this were male patients from Norway, with a borderline statistically increased OR (1.17, 95% CI 1.00–1.37), men from Finland with a slightly decreased OR (0.88, 95% CI 0.81–0.95), and female patients from Iran with an increased OR (1.39, 95% CI 1.00–1.93) (Table S1&S2 in File S1).

The likelihood of treatment at a CCU decreased with increasing age at diagnosis of first-time MI, regardless of sex and country of birth. The exception to this trend was patients younger than 35 years who also had a low chance of CCU admission (Table 4).

**Table 4:** Odds ratio (OR) and 95% confidence interval (CI) of access to coronary care units (CCUs) by age at diagnosis, hospital characteristics, and medical conditions as well as by sex and country of birth.

	Men				Women			
	Sweden –born		Foreign –born		Sweden –born		Foreign –born	
	Access to CCU yes/no	OR (95% CI)	Access to CCU yes/no	OR (95% CI)	Access to CCU yes/no	OR (95% CI)	Access to CCU yes/no	OR (95% CI)
**Age at diagnosis, years**								
<35	175/107	**3.22 (2.48–4.17)**	51/17	**5.05 (2.64–9.69)**	65/56	**2.99 (2.06–4.33)**	10/5	**7.20 (2.37–21.8)**
35–39	393/96	**7.97 (6.25–10.17)**	154/31	**8.23 (4.93–13.8)**	149/37	**9.37 (6.44–13.6)**	26/8	**10.44 (4.49–24.3)**
40–44	1,032/225	**8.45 (7.15–9.98)**	477/94	**8.05 (5.38–12.0)**	391/89	**9.44 (7.40–12.0)**	90/21	**12.80 (7.44–22.0)**
45–49	2,296/474	**9.04 (7.96–10.3)**	837/156	**8.61 (5.91–12.6)**	692/221	**7.10 (6.00–8.40)**	168/55	**8.78 (5.92–13.0)**
50–54	4,389/954	**8.56 (7.71–9.51)**	1,178/242	**8.10 (5.64–11.7)**	1,265/386	**7.17 (6.28–8.19)**	291/91	**9.19 (6.54–12.9)**
55–59	72,83/1,668	**8.25 (7.52–9.05)**	1,380/355	**6.97 (4.89–9.94)**	2,121/680	**6.99 (6.28–7.78)**	399/143	**8.58 (6.31–11.6)**
60–64	8,795/2,379	**7.21 (6.61–7.88)**	1,448/427	**6.38 (4.49–9.05)**	3,066/1,104	**6.29 (5.74–6.91)**	568/232	**7.51 (5.65–9.99)**
65–69	8,665/2,858	**6.38 (5.86–6.96)**	1,356/462	**6.11 (4.31–8.65)**	3,813/1,571	**5.79 (5.32–6.30)**	762/327	**7.84 (5.98–10.3)**
70–74	8,937/3,939	**5.27 (4.85–5.74)**	1,257/565	**5.24 (3.71–7.40)**	5,081/2,632	**4.86 (4.51–5.24)**	945/460	**7.16 (5.52–9.29)**
75–79	9,278/5,601	**4.25 (3.92–4.61)**	968/584	**4.26 (3.01–6.02)**	7,044/4,692	**4.04 (3.77–4.32)**	1,052/696	**5.68 (4.41–7.32)**
80–84	8,392/7,174	**3.25 (3.00–3.52)**	634/514	**3.31 (2.33–4.70)**	8,042/8,053	**2.87 (2.69–3.06)**	846/857	**3.97 (3.09–5.10)**
85–89	4,349/6,312	**1.94 (1.79–2.11)**	219/296	**1.98 (1.36–2.89)**	5,743/9,350	**1.82 (1.71–1.94)**	387/650	**2.47 (1.89–3.21)**
≥90	1,231/3,482	1	1/150	1	2,273/7,302	1	95/417	1
**Hospital characteristics**								
Without CCU	13,701/7,405	**0.92 (0.86–0.98)**	2,284/811	1.06 (0.89–1.26)	8,421/8,045	**0.89 (0.83–0.96)**	1,373/965	0.93 (0.78–1.11)
With CCU	51,514/27,864	1	7,726/3,082	1	31,324/28,128	1	4,266/2,997	1
**Medical conditions**								
Diabetes								
Yes	14,614/8,468	**0.93 (0.90–0.97)**	2,608/1,132	0.94 (0.86–1.03)	9,409/8,012	0.99 (0.95–1.03)	1,693/1,137	1.01 (0.91–1.12)
No	50,601/26,801	1	7,402/2,761	1	30,336/28,161	1	3,946/2,825	1
Hypertension								
Yes	30,276/14,733	**1.15 (1.11–1.18)**	4,556/1,747	1.08 (0.99–1.17)	21,677/17,469	**1.21 (1.17–1.25)**	3,335/2,082	**1.22 (1.11–1.34)**
No	34,939/20,536	1	5,454/2,146	1	18,068/18,704	1	2,304/1,880	1
Hyperlipidemia								
Yes	20,041/5,524	**1.66 (1.60–1.72)**	3,623/914	**1.49 (1.36–1.64)**	9,596/3,497	**1.88 (1.79–1.97)**	1,792/604	**1.78 (1.59–2.00)**
No	45,174/29,745	1	6,387/2,979	1	30,149/32,676	1	3,847/3,358	1
Strok:								
Yes	6,350/6,860	**0.64 (0.61–0.67)**	796/626	**0.69 (0.61–1.78)**	4,538/6,935	**0.66 (0.63–0.69)**	623/701	**0.69 (0.61–0.78)**
No	58,865/28,409	1	9,214/3,267	1	35,207/29,238	1	5,016/3,261	1
Heart failure								
Yes	4,264/6,625	**0.56 (0.54–0.59)**	633/707	**0.60 (0.53–0.69)**	4,015/7,643	**0.69 (0.66–0.72)**	602/933	**0.63 (0.55–0.72)**
No	60,951/28,644	1	9,377/3,186	1	35,730/28,530	1	5,037/3,029	1
Angina								
Yes	9,299/7,605	**0.77 (0.74–0.80)**	1,485/956	**0.71 (0.64–0.79)**	5,899/6,890	**0.93 (0.89–0.97)**	997/959	**0.79 (0.70–0.88)**
No	55,916/27,664	1	8,525/2,937	1	33,846/29,283	1	4,642/3,003	1
Atrial fibrillation								
Yes	5,259/5,927	**0.81 (0.77–0.85)**	558/525	**0.75 (0.64–0.86)**	4,170/6,529	**0.87 (0.83–0.91)**	541/656	0.91 (0.79–1.04)
No	59,956/29,342	1	9,452/3,368	1	35,575/29,644	1	5,098/3,306	1
Pulmonary embolism								
Yes	583/562	**0.82 (0.72–0.93)**	57/47	0.78 (0.51–1.20)	499/746	**0.78 (0.69–0.88)**	54/61	0.86 (0.58–1.28)
No	64,632/34,707	1	9,953/3,846	1	39,246/35,427	1	5,585/3,901	1
COLγ								
Yes	2,476/3,031	**0.64 (0.60–0.68)**	426/389	**0.69 (0.59–0.81)**	2,164/3,003	**0.64 (0.60–0.68)**	370/453	**0.63 (0.54–0.74)**
No	62,739/32,238	1	9,584/3,504	1	37,581/33,170	1	5,269/3,509	1
Cancer:								
Yes	11,328/9,266	**0.78 (0.75–0.81)**	1,330/850	**0.76 (0.68–0.85)**	8,375/8,553	**0.84 (0.81–0.88)**	1,065/901	**0.79 (0.71–0.88)**
No	53,887/26,003	1	8,680/3,043	1	31,370/27,620	1	4,574/3,061	1

γ Chronic obstructive lung disease.

ORs of reference groups were set as 1.

All ORs for presence of medical conditions (yes) were compared with absence of the same characteristic (no).

All ORs were mutually adjusted for education, age at diagnosis, medical conditions, availability of CCUs, and county of diagnosis when applicable.

**Bold** numbers indicate statistically significant ORs.

The likelihood of treatment at a CCU was about 10% higher for Sweden-born men and women initially treated in hospitals with CCU facilities than for patients initially treated for their MI at a hospital without a CCU. No similar pattern was observed for foreign-born MI patients (Table 4).

First-time MI patients with hyperlipidemia or hypertension, irrespective of sex and country of birth, had a higher access to the CCU. By contrast, patients with a medical condition of stroke, heart failure, angina, atrial fibrillation, pulmonary embolism, chronic obstructive lung disease, or cancer were less likely to be admitted to the CCU (Table 4).

## Discussion

In this nationwide study, despite an increasing proportion being treated at a CCU for first MI during the study period between 2001 and 2009, we found a lower admission among women compared with men and among poorly educated compared with highly educated patients irrespective of country of birth (within versus outside Sweden). The observed equality in CCU treatment after first-time MI between foreign-born and Sweden-born patients in this study is novel and, to the best of our knowledge, has not been reported previously. Additionally, this is the first nationwide study to investigate the equality of access to the CCU covering the whole population of Sweden, including a large foreign-born population, utilizing high-quality Swedish registers such as RIKS-HIA with recorded information on consecutive patients admitted to CCUs in almost all hospitals throughout the country over the last 10 years.

We believe that the long-term trends in CCU treatment after first-time MI have not previously been studied at a national level. The observed increase in CCU treatment over time in our study could be an indication of an overall quality improvement in coronary care management [12], [13]. The quality of classification of first-time MI cases and CCU admissions is high in this study. From 2001 onwards, the Swedish National Patient Register included information on hospitalized patients as well as outpatient visits to specialist care and day care visits to hospitals throughout Sweden. It has been shown that it is reasonable to identify MI cases [14] and coronary risk factors [15] in this way. The information on diagnosis has been validated and in general found to be of high quality, particularly suitable for large-scale population-based research with long follow-up [16]. In 2010, based on 132 reviewed papers, the positive predict value was found about 85% to 95% [16]. The drop-out rate for 2007 has been estimated to less than one percent [17].The coverage of CCU admissions by means of RIKS-HIA is high and is estimated to be at least 95% of all admissions [13], [18].

The lower rate of treatment at a CCU among women compared with men observed in this study has previously been reported from U.S., U.K. and Italy [2], [7], [19], [20] and from small regional studies in Sweden [21], [22], [23]. We were able to confirm this finding even after taking into account age, educational level as an indication of SEP, country of birth, and a number of other potential confounders. One possible explanation for this sex difference is that the predominant symptoms of MI in women may be different from those in men. It has been reported that women have a lower prevalence of chest pain, which is a hallmark symptom among men [24]. Instead female MI patients have a higher prevalence of fatigue, neck pain, syncope, nausea, right arm pain, dizziness, jaw pain, shortness of breath, and weakness [24], [25]. Women also tend to have a different attitude when seeking medical care including delaying consultation with medical care [26]. This is important and should be realized by healthcare specialists as it may result in patient- and healthcare-related delays in the diagnosis of MI to a stage when referral to the CCU is not feasible.

In contrast to the findings of a number of similar studies conducted in countries other than Sweden [3], [4], [8], [27], we observed equality of access to the CCU between Sweden-born and foreign-born patients both when considered as one group and when categorized by individual country of birth. Further, we found that the equality was evident across all time periods studied. The free healthcare system for all residents in Sweden may partly explain the equal ability to access the CCU for foreign-born and Sweden-born MI patients. We adjusted for education in all the analyses. However, the fact that a larger proportion of foreign-born than Sweden-born patients have a high level of education could also in part explain the observed equal access to the CCU between individuals born within and outside Sweden, as a result of residual confounding by education.

The lower likelihood of being treated at a CCU among patients with a low level of education compared with those with a higher educational level found in this study across both sexes and irrespective of country of birth was not explained by medical conditions. Other unexplored factors such as better communication, better health awareness and higher demand among highly educated patients might affect whether or not physicians decide to admit to the CCU. The three social dimensions (education, occupation and income) have been shown to affect myocardial infarction, even when the effect of the other two dimensions is controlled for [28]. Since immigrants may have completed their education in their native countries but still be underemployed in their new country, therefore leading to a lower level of SEP. Education level is more relevant and a better proxy for SEP and in the context of this study than employment status and income.

The increased likelihood of being treated at a CCU among patients with hyperlipidemia or hypertension observed in this study was expected. By contrast, the decreased access for patients with a history of other medical conditions, including diabetes, was somewhat surprising as patients with comorbidities should have a greater potential need for treatment at a CCU. However, difficulties in identifying MI-related symptoms among all other non-specific symptoms in these patients might result in delays in diagnosis of MI and thus referral to a CCU.

We were not able to monitor any MI events that occurred in a foreign-born individual in their country of birth. Thus, we assumed that the first record of an MI in the Swedish health registers was the first MI for all individuals. Hence, it is possible, mostly among foreign-born patients, that some second-time MIs may have been misclassified as first-time MI. This could have resulted in overestimation of the number of true first-time MIs and underestimation of the prevalence of certain similar medical conditions among foreign-born men and women. We addressed this issue of misclassification by restricting the cohort to foreign-born individuals living in Sweden for more than 14 years. The results in the restricted and entire cohorts were essentially the same. We lacked information on MI severity as a potential confounding factor in this study. To overcome this limitation we restricted analysis to patients with first MI who survived the first day of hospital stay; this yielded similar results compared with the entire cohort.

We lacked information on MI type; ST-segment elevation (STEMI) or non-ST-segment elevation (NSTEMI). Although, all hospitals are required to follow the same protocol in treating MI patients regardless of MI type in Sweden, delays from symptom onset to hospital presentation for NSTEMI patients have been observed [29]. It could be speculated upon that the observed lower likelihood of being treated at CCU among women might partly be explained by proportionally more NSTEMI patients among women than men [30] [29] [27].

Information on practice patterns and management protocols for referral to CCU across different hospitals in Sweden was not available in our study. There are national guidelines but they don’t specify the indication for CCU care. Heterogeneity in treatment practices such as parenteral anticoagulants, intravenous B-blockade, intravenous nitroglycerin, use of echocardiography and early revascularization across hospitals has been observed in Sweden [31]. However, it is unlikely that these differences would interact with sex. And, we adjusted the hospital type in our model to reduce the bias caused.

The results of this study suggest that the Swedish healthcare system provides equal access to CCUs for foreign-born and Sweden-born first-time MI patients. Despite an increasing proportion receiving CCU treatment during the study period, important inequalities were still evident as a lower likelihood for treatment at a CCU for women, elderly patients, those with a low level of education (a marker of SEP), and those with a history of certain medical conditions. These findings suggest that the healthcare authorities should continue efforts to abolish these remaining inequalities.

## Supporting Information

File S1 **Table S1 & S2.** Table S1. Odds ratio (OR) and 95% confidence interval (CI) of access to coronary care units (CCUs) by continent, subregion, and country for men living in Sweden between 2001 and 2009. Table S2. Odds ratio (OR) and 95% confidence interval (CI) of access to coronary care units (CCUs) by continent, subregion, and country for women living in Sweden between 2001 and 2009.
(DOC)
