# Supplementary material for: Likelihood of Treatment in a Coronary Care Unit for a First-Time Myocardial Infarction in Relation to Sex, Country of Birth and Socioeconomic Position in Sweden
Source: PLoS One. 2013 Apr 25;8(4):e62316. doi: 10.1371/journal.pone.0062316 (PMC3636189; doi:10.1371/journal.pone.0062316)
Supplement: File S1 — Table S1 & S2. Table S1. Odds ratio (OR) and 95% confidence interval (CI) of access to coronary care units (CCUs) by continent, subregion, and country for men living in Sweden between 2001 and 2009. Table S2. Odds ratio (OR) and 95% confidence interval (CI) of access to coronary care units (CCUs) by continent, subregion, and country for women living in Sweden between 2001 and 2009. (DOC) [file pone.0062316.s001.doc]

| **Table S1: Odds ratio (OR) and 95% confidence interval (CI) of access to coronary care units (CCUs) by continent, subregion, and country for men living in Sweden between 2001 and 2009** | | | | | | | | | | |
| --- | --- | --- | --- | --- | --- | --- | --- | --- | --- | --- |
|  | | **Access to CCU** | |  | |  | | **Access to CCU** | |  |
| **Country of birth** | | **Yes/No** | | **OR(95% CI)** | | **Country of birth** | | **Yes/No** | | **OR(95% CI)** |
| **Sweden** | | 65,215/35,269 | | 1 | | United Kingdom | | 132/31 | | 1.37(0.91–2.06) |
| **Africa** | | 247/72 | | 0.92(0.71–1.21) | | Estonia | | 172/125 | | 0.97(0.75–1.25) |
| **Northern Africa** | | 97/30 | | 0.90(0.59–1.36) | | Norway | | 562/272 | | **1.17(1.00–1.37)** |
| Algeria | | 10/3 | | 0.85(0.23–3.19) | | Denmark | | 633/333 | | 1.08(0.93–1.24) |
| Tunisia | | 24/6 | | 1.07(0.43–2.67) | | Finland | | 2,464/1,100 | | **0.88(0.81–0.95)** |
| Egypt | | 29/9 | | 0.83(0.39–1.77) | | **Southern Europe** | | 1,655/521 | | 1.10(0.99–1.23) |
| Morocco | | 29/12 | | 0.75(0.38–1.49) | | Serbia | | 9/3 | | 0.89(0.23–3.50) |
| **Southern Africa** | | 8/4 | | 0.61(0.18–2.10) | | Slovenia | | 11/2 | | 1.74(0.38–8.04) |
| South Africa | | 8/4 | | 0.61(0.18–2.10) | | Albania | | 8/2 | | 1.87(0.36–9.82) |
| **Western Africa** | | 34/11 | | 0.82(0.41–1.64) | | Portugal | | 16/7 | | 0.91(0.36–2.29) |
| Gambia | | 17/1 | | 3.84(0.51–28.9) | | Macedonia | | 24/15 | | 0.53(0.27–1.04) |
| Uganda | | 7/3 | | 0.70(0.18–2.81) | | Croatia | | 48/14 | | 1.05(0.57–1.93) |
| **Eastern Africa** | | 99/22 | | 1.17(0.73–1.87) | | Spain | | 59/12 | | 1.78(0.93–3.39) |
| Kenya | | 5/1 | | 1.15(0.13–9.91) | | Greece | | 137/36 | | 1.18(0.80–1.73) |
| Tanzania | | 7/2 | | 0.75(0.15–3.63) | | Italy | | 135/55 | | 1.09(0.79–1.52) |
| Eritrea | | 19/7 | | 0.67(0.28–1.63) | | Bosnia | | 413/125 | | 1.22(0.99–1.51) |
| Somalia | | 37/7 | | 1.62(0.70–3.72) | | **Western Europe** | | 773/317 | | 1.14(0.99–1.31) |
| Ethiopia | | 30/5 | | 1.43(0.54–3.75) | | Belgium | | 9/2 | | 2.32(0.46–11.5) |
| **Central Africa** | | 9/5 | | 0.52(0.17–1.60) | | Switzerland | | 26/11 | | 1.19(0.55–2.57) |
| **Other^γ^** | | 25/12 | | 0.59(0.29–1.19) | | France | | 43/14 | | 1.16(0.61–2.20) |
| **Asia** | | 2,041/572 | | 1.07(0.96–1.18) | | Netherlands | | 41/19 | | 1.03(0.58–1.84) |
| **Eastern Asia** | | 40/22 | | 0.96(0.55–1.69) | | Austria | | 105/41 | | 1.26(0.86–1.85) |
| Korean Republic | | 5/2 | | 0.92(0.13–6.75) | | Germany | | 548/230 | | 1.11(0.94–1.31) |
| China | | 32/16 | | 1.18(0.63–2.24) | | **Eastern Europe** | | 918/406 | | 1.05(0.93–1.19) |
| **Southern Asia** | | 694/158 | | **1.23(1.02–1.47)** | | Ukraine | | 7/2 | | 1.39(0.27–7.01) |
| Sri Lanka | | 22/4 | | 1.49(0.51–4.39) | | Russia | | 17/7 | | 1.18(0.47–3.01) |
| Afghanistan | | 41/9 | | 1.34(0.64–2.82) | | Bulgaria | | 22/6 | | 1.30(0.51–3.32) |
| Bangladesh | | 47/10 | | 1.11(0.55–2.24) | | Romania | | 94/40 | | 1.07(0.72–1.60) |
| Pakistan | | 59/11 | | 1.22(0.64–2.34) | | Former Czechoslovakia* | | 107/51 | | 0.99(0.69–1.42) |
| India | | 81/16 | | 1.61(0.92–2.80) | | Former Soviet Union† | | 73/50 | | 0.98(0.66–1.45) |
| Iran | | 435/108 | | 1.14(0.92–1.43) | | Hungary | | 262/100 | | 1.14(0.89–1.45) |
| **Southeast Asia** | | 61/22 | | 0.78(0.47–1.28) | | Poland | | 331/149 | | 1.01(0.82–1.25) |
| Philippines | | 10/1 | | 2.88(0.35–23.4) | | Former Yugoslavia^$^ | | 794/250 | | 1.05(0.90–1.21) |
| Indonesia | | 11/3 | | 1.28(0.35–4.75) | | **Other^γ^** | | 14/5 | | 1.54(0.50–4.76) |
| Vietnam | | 30/10 | | 0.85(0.41–1.76) | | **Latin America** | | 214/53 | | 1.27(0.93–1.74) |
| **Western Asia** | | 1,246/370 | | 1.02(0.90–1.16) | | **South America** | | 187/45 | | 1.05(0.93–1.19) |
| Yemen | | 5/2 | | 0.55(0.11–2.89) | | Colombia | | 8/2 | | 1.65(0.31–8.78) |
| Azerbaijan | | 6/2 | | 0.81(0.16–4.24) | | Brazil | | 7/2 | | 0.67(0.14–3.27) |
| Armenia | | 8/3 | | 0.80(0.20–3.14) | | Peru | | 11/4 | | 0.75(0.22–2.52) |
| Cyprus | | 9/1 | | 1.95(0.25–15.4) | | Uruguay | | 15/4 | | 0.99(0.31–3.15) |
| Israel | | 10/2 | | 1.59(0.32–8.01) | | Argentina | | 22/4 | | 1.69(0.57–5.03) |
| Jordan | | 18/2 | | 2.40(0.54–10.6) | | Chile | | 118/26 | | 1.43(0.92–2.22) |
| Palestinian territories | | 36/19 | | 0.60(0.34–1.08) | | **Central America** | | 8/5 | | 0.63(0.20–1.99) |
| Lebanon | | 158/40 | | 1.03(0.72–1.48) | | El Salvador | | 6/2 | | 1.30(0.25–6.72) |
| Syria | | 148/51 | | 0.92(0.66–1.29) | | **Caribbean** | | 19/3 | | 2.35(0.65–8.44) |
| Iraq | | 485/128 | | 1.11(0.91–1.37) | | Cuba | | 10/1 | | 3.89(0.43–35.1) |
| Turkey | | 356/116 | | 1.04(0.83–1.30) | | **Other^γ^** | | 17/8 | | 0.76(0.31–1.82) |
| **Other^γ^** | | 29/16 | | **0.45(0.24–0.85)** | | **Northern America** | | 127/61 | | 1.27(0.91–1.76) |
| **Europe** | | 7,369/3,132 | | 1.01(0.97–1.06) | | Canada | | 12/6 | | 1.12(0.40–3.13) |
| **Nothern Europe** | | 4,023/1,888 | | 0.96(0.90–1.02) | | USA | | 115/55 | | 1.29(0.91–1.83) |
| Iceland | | 25/7 | | 1.07(0.44–2.58) | | **Oceania** | | 9/2 | | 1.43(0.27–7.49) |
| Latvia | | 28/23 | | 0.69(0.38–1.26) | |  | |  | |  |
| All odds ratios were adjusted for age, level of education, year of diagnosis, medical conditions, and availability of CCU facitilties.  **γ** Countries with fewer than five cases of MI were grouped together.  *The former Czechoslovakia includes the Czech Republic and Slovakia.  † The former Soviet Union includes Belarus, Moldova, Russia, and Ukraine.  ^$^ The former Yugoslavia includes Croatia, Macedonia, Serbia, Slovenia, and Montenegro.  Bold numbers indicate statistically significant ORs. | | | | | | | | | | |
| **Table S2: Odds ratio (OR) and 95% confidence interval (CI) of access to coronary care units (CCUs) by continent, subregion, and country for women living in Sweden between 2001 and 2009** | | | | | | | | | | |
|  | **Access to CCU** | |  | |  | | **Access to CCU** | |  | |
| **Country of birth** | **Yes/No** | | **OR(95% CI)** | | **Country of birth** | | **Yes/No** | | **OR(95% CI)** | |
| **Sweden** | 39,745/36,173 | | 1 | | Finland | | 1,927/1,302 | | 0.93(0.87–1.01) | |
| **Africa** | 48/30 | | 0.75(0.46–1.22) | | **Southern Europe** | | 690/397 | | 1.02(0.89–1.17) | |
| **Northern Africa** | 12/10 | | 0.85(0.35–2.11) | | Serbia | | 8/7 | | 0.35(0.12–1.05) | |
| **Western Africa** | 9/4 | | 0.74(0.22–2.47) | | Portugal | | 6/4 | | 0.82(0.22–3.07) | |
| **Eastern Africa** | 24/14 | | 0.71(0.36–1.42) | | Macedonia | | 7/4 | | 1.29(0.34–4.79) | |
| Eritrea | 5/3 | | 0.60(0.14–2.59) | | Croatia | | 15/7 | | 1.30(0.51–3.28) | |
| Somalia | 11/5 | | 1.25(0.41–3.78) | | Spain | | 16/18 | | 0.61(0.28–1.33) | |
| Ethiopia | 5/5 | | 0.33(0.09–1.17) | | Greece | | 36/22 | | 0.79(0.45–1.38) | |
| **Other^γ^** | 22/16 | | 0.68(0.34–1.37) | | Italy | | 24/20 | | 1.02(0.54–1.93) | |
| **Asia** | 657/330 | | **1.19(1.03–1.38)** | | Bosnia | | 268/147 | | 1.17(0.94–1.46) | |
| **Eastern Asia** | 17/15 | | 0.89(0.43–1.87) | | **Western Europe** | | 464/398 | | 0.91(0.79–1.05) | |
| Japan | 6/1 | | 2.93(0.33–25.7) | | Belgium | | 5/3 | | 1.24(0.26–5.82) | |
| China | 10/13 | | 0.67(0.28–1.59) | | Switzerland | | 5/7 | | 0.55(0.17–1.83) | |
| **Southern Asia** | 194/88 | | 1.25(0.95–1.63) | | France | | 16/12 | | 1.21(0.54–2.72) | |
| Sri Lanka | 10/4 | | 1.21(0.37–3.95) | | Netherlands | | 24/10 | | 1.89(0.87–4.13) | |
| Afghanistan | 15/6 | | 1.14(0.43–3.04) | | Austria | | 42/45 | | 0.73(0.46–1.15) | |
| Pakistan | 11/2 | | 1.98(0.43–9.19) | | Germany | | 372/321 | | 0.90(0.77–1.05) | |
| India | 23/14 | | 0.85(0.42–1.74) | | **Eastern Europe** | | 630/498 | | 1.01(0.89–1.15) | |
| Iran | 128/58 | | **1.39(1.00–1.93)** | | Ukraine | | 5/3 | | 1.63(0.36–7.33) | |
| **Southeastern Asia** | 38/15 | | 1.34(0.71–2.51) | | Russia | | 14/16 | | 0.85(0.40–1.81) | |
| Thailand | 11/3 | | 1.45(0.38–5.57) | | Bulgaria | | 12/4 | | 2.69(0.80–9.04) | |
| Philippines | 8/1 | | 3.45(0.43–27.8) | | Romania | | 54/31 | | 1.37(0.85–2.20) | |
| Vietnam | 10/9 | | 0.76(0.29–1.97) | | Former Czechoslovakia* | | 62/51 | | 0.93(0.63–1.39) | |
| **Western Asia** | 408/212 | | 1.18(0.98–1.42) | | Former Soviet Union† | | 81/96 | | 0.84(0.61–1.15) | |
| Palestinian territories | 11/16 | | 0.46(0.21–1.05) | | Hungary | | 109/73 | | 1.17(0.85–1.61) | |
| Lebanon | 40/14 | | 1.42(0.74–2.70) | | Poland | | 292/221 | | 0.99(0.82–1.19) | |
| Syria | 62/27 | | 1.30(0.80–2.09) | | Former Yugoslavia^$^ | | 306/171 | | 1.00(0.82–1.23) | |
| Iraq | 147/74 | | 1.18(0.87–1.59) | | **Other^γ^** | | 6/5 | | 0.57(0.17–1.92) | |
| Turkey | 140/78 | | 1.28(0.95–1.73) | | **Latin America** | | 92/57 | | 1.04(0.73–1.48) | |
| **Other^γ^** | 25/10 | | 1.02(0.47–2.22) | | **South America** | | 86/51 | | 1.07(0.74–1.56) | |
| **Europe** | 4,744/3,455 | | 0.97(0.93–1.02) | | Brazil | | 11/1 | | 5.47(0.68–43.7) | |
| **Nothern Europe** | 2,960/2,162 | | 0.97(0.91–1.03) | | Uruguay | | 6/2 | | 3.27(0.60–17.8) | |
| Lithuania | 6/3 | | 1.72(0.40–7.30) | | Argentina | | 7/4 | | 1.85(0.48–7.21) | |
| Iceland | 6/7 | | 0.52(0.16–1.67) | | Chile | | 54/35 | | 0.96(0.61–1.52) | |
| Latvia | 26/37 | | 0.78(0.45–1.34) | | **Other^γ^** | | 14/15 | | 0.54(0.25–1.17) | |
| United Kingdom | 30/19 | | 1.17(0.62–2.20) | | **Northern America** | | 93/84 | | 1.24(0.90–1.70) | |
| Estonia | 127/143 | | 0.86(0.67–1.11) | | Canada | | 9/8 | | 1.01(0.36–2.82) | |
| Norway | 499/391 | | 1.11(0.97–1.28) | | USA | | 84/76 | | 1.27(0.91–1.77) | |
| Denmark | 338/260 | | 1.00(0.84–1.18) | |  | |  | |  | |
| All odds ratios were adjusted for age, level of education, year of diagnosis, medical conditions, and availability of CCU facilities.  **γ**Countries with fewer than five cases of MI were grouped together.  *The former Czechoslovakia includes the Czech Republic and Slovakia.  † The former Soviet Union includes Belarus, Moldova, Russia, and Ukraine.  ^$^  The former Yugoslavia includes Croatia, Macedonia, Serbia, Slovenia, and Montenegro.  Bold numbers indicate statistically significant ORs. | | | | | | | | | | |
